# Thyroid volumes and serum VEGF levels in dyslipidemic patients: effects of statin treatment

**DOI:** 10.3906/sag-1708-106

**Published:** 2019-06-18

**Authors:** Kadriye AYDIN, Neşe ERSÖZ GÜLÇELİK, Murat TUNCEL, Cafer BALCI, Şafak AKIN, Neşe ÇINAR, Firuzan FIRAT, Meltem ÇAĞLAR, Aydan USMAN, Alper GÜRLEK

**Affiliations:** 1 Department of Endocrinology and Metabolism, Kartal Dr. Lütfi Kırdar Training and Research Hospital,University of Health Sciences, İstanbul Turkey; 2 Department of Endocrinology and Metabolism, Ankara Gülhane Medical School, Ankara Turkey; 3 Department of Nuclear Medicine, Faculty of Medicine, Hacettepe University, Ankara Turkey; 4 Department of Internal Medicine, Faculty of Medicine, Hacettepe University, Ankara Turkey; 5 Department of Endocrinology and Metabolism, Faculty of Medicine, Hacettepe University, Ankara Turkey

**Keywords:** Statin, vascular endothelial growth factor, thyroid nodule, dyslipidemia

## Abstract

**Background/aim:**

Defective vascularization may be important in thyroid nodular disease. In this study, we aimed to investigate serum vascular endothelial growth factor (VEGF) levels in dyslipidemic patients with thyroid nodules, as well as the effects of statin therapy.

**Materials and methods:**

The study included 37 dyslipidemic patients with thyroid nodules and 32 dyslipidemic patients without thyroid nodules. Anthropometry, serum VEGF levels, biochemical parameters, thyroid-stimulating hormone (TSH), free triiodothyronine (fT3) and free thyroxine (fT4) levels, and thyroid sonography were determined before and after 6 months of statin therapy.

**Results:**

Patients with and without thyroid nodules had similar metabolic parameters. Serum VEGF levels did not differ between the groups. In patients with nodules, VEGF levels remained unchanged (P = 0.931) after statin therapy. However, serum VEGF levels were lowered by statin treatment in patients without nodules (P = 0.030). Statin therapy resulted in a decrease in the dominant thyroid nodule volume. The changes in thyroid volume and dominant thyroid nodule volume were not correlated with changes in VEGF, body mass index, total cholesterol, low-density lipoprotein cholesterol, or homeostatic model assessment of insulin resistance (HOMA-IR).

**Conclusion:**

Although statin treatment decreases serum VEGF levels in dyslipidemic patients without thyroid nodules, it has no lowering effect on serum VEGF levels in patients with thyroid nodules. The decrease in thyroid nodule volume with statin treatment was associated with neither metabolic parameters nor serum VEGF levels.

## 1. Introduction

Thyroid hormones appear to serve as a general metabolic controller coordinating many metabolic processes; thus, the association of metabolic syndrome and its components with thyroid function and nodule formation is an intriguing area of research in thyroidology (1–3). In a number of studies, increased thyroid volume and nodularity were shown to be associated with metabolic syndrome parameters, including dyslipidemia (1–3). 

Vascularization is an important feature of tumor growth and might promote the progression of thyroid nodules. Recently, Wang et al. demonstrated that insulin resistance and hyperglycemia resulted in increased intranodular vascularization, which might contribute to the growth and progression of nodules (4). Vascular endothelial growth factor (VEGF) is a critical regulator of angiogenesis and is involved in tumor development. VEGF expression was found to be associated with lymph node metastasis, advanced tumor stage, and increased recurrence risk in malignant thyroid cancers (5–7). Research investigating the role of VEGF in benign thyroid disorders yielded controversial results. In vitro and animal studies have revealed the presence of VEGF in follicular cells from normal thyroids and VEGF is upregulated in benign thyroid disorders (8,9). Other studies found no or weak expression of VEGF in benign nodular goiter (10,11). Malkomes et al. recently demonstrated coexpression of VEGF and its receptors in normal thyrocytes and in benign thyroid diseases, measuring higher concentrations of VEGF protein in nodular tissue (12). Itoh et al. found that there was no VEGF expression in normal thyroid tissue, slightly positive expression in benign follicular tumors, and increased expression in differentiated thyroid cancers; they suggested that VEGF expression reflects both transformation and differentiation states of thyroid tumors (13). 

Statins are cholesterol-lowering agents that prevent reduction of 3-hydroxy-3-methylglutaryl coenzyme A (HMG-CoA) to mevalonate by inhibiting HMG-CoA reductase. Beyond lipid-lowering effects, they have immunomodulatory, antiinflammatory, and antioxidant activity unrelated to the effects on lipid metabolism (14–17). Furthermore, statins have a potential role in angiogenesis, a process intimately linked with angiogenic growth factors acting on the endothelium. Statins were demonstrated to reduce VEGF expression or serum VEGF levels in a variety of diseases with increased VEGF expression such as type 2 diabetes, coronary artery disease, dyslipidemia, and lung cancer, and their use has been encouraged for their pleiotropic and antiangiogenic effects (17–19). 

Moreover, the antigoitrogenic effects of statins have been demonstrated in a number of in vitro studies, which have revealed that statins induce apoptosis in thyroid cells and decrease thyroid hypertrophy and hyperplasia induced by goitrogenic agents (20–22). The antigoitrogenic effect of statins was further evaluated by a retrospective analysis confirming that treatment with statins was associated with smaller thyroid size and significantly lower prevalence, number, and volume of thyroid nodules (23). 

Dyslipidemia is shown to be associated with increased thyroid volume and thyroid nodules. Statins have contingent beneficial effects on thyroid nodules and a lowering effect on VEGF levels in a variety of VEGF-increased conditions. Therefore, we designed this study to evaluate serum VEGF levels in dyslipidemic patients with and without thyroid nodules and prospectively evaluate the effect of statin therapy on thyroid nodules and serum VEGF levels. 

## 2. Materials and methods

We enrolled 37 dyslipidemic patients with thyroid nodules and 32 dyslipidemic patients without thyroid nodules who were intended to receive statin treatment according to Adult Treatment Panel III (ATP-III) criteria. All the patients were evaluated prior to statin therapy and after 6 months of statin therapy. Other medications remained unchanged during the study period. Thyroid ultrasonographic evaluation, detailed physical examination including anthropometric measurements and blood pressure, analysis of lipid parameters, fasting blood glucose and insulin, thyroid-stimulating hormone (TSH), free triiodothyronine (fT3) and free thyroxine (fT4) levels, thyroglobulin, antithyroid peroxidase antibody (anti-TPO), antithyroglobulin antibody (anti-TG), and VEGF levels were obtained before and after therapy. Body mass index (BMI) was calculated according to the following formula: weight in kilograms/square of height in meters. Waist circumference was measured at the level of the umbilicus; hip circumference was measured at the largest point. Waist to hip ratio (WHR) was calculated. Patients were evaluated at 2-month intervals to optimize the low-density lipoprotein cholesterol (LDL-C) goals, and drug dosages were titrated accordingly. After 6 months of treatment with statin, subjects were asked to return all empty blister packs to determine the individual’s compliance. Patients with malignant disease, renal or hepatic disease, acute or chronic infection, rheumatologic disorders, and any medication affecting thyroid function tests were excluded from the study, as well as patients who did not complete the 6-month course of therapy due to side effects or lack of follow-up. The study protocol was approved by the university’s local ethics committee and was performed in accordance with the Declaration of Helsinki. All participants provided written informed consent.

### 2.1. Ultrasonographic evaluation

Ultrasonographic thyroid evaluations were performed by the same skilled sonographer using a Doppler ultrasonographic scanner (LOGIQ 3, General Electric, Solingen, Germany) equipped with a 10-MHz linear transducer. Thyroid volume, number of thyroid nodules, sum of thyroid nodule volumes, dominant nodule volume, and characteristic sonographic features of each nodule were recorded. The reproducibility of ultrasonographic measurements was tested in a control group consisting of 10 healthy volunteers with measurements performed by the same operator 3 times at 10-min intervals and analyzed using analysis of variance. The coefficient of variation was 4.1%.

The volumes of thyroid glands and thyroid nodules were calculated with the standard formula for an ellipsoid volume, and the thyroid volume was the sum of all thyroid lobes’ volumes. In cases of multinodularity, sonographic features of up to 4 nodules in each lobe were evaluated. Sonographic characteristics of nodules were evaluated in 5 categories: (a) structure: solid, cystic, or mix type; (b) echogenicity: isoechoic, hypoechoic, or hyperechoic; (c) vascularity: internal, peripheral, both, or none; (d) border shape: regular or irregular; (e) halo: hypoechoic, hyperechoic, or none. Differences in these characteristics for each nodule after 6 months of therapy were recorded. 

### 2.2. Laboratory analysis

Blood samples were obtained after an overnight fast in the early morning between 0800 and 1000 hours. The sera of blood samples centrifuged at 4000 rpm for 20 min were stored at –80 °C until assayed. Triglycerides (TG), total cholesterol (TC), LDL-C, and high-density lipoprotein cholesterol (HDL-C) were measured using enzymatic calorimetric kits with intra- and interassay coefficients of variation (CVs) of <10% (Roche Diagnostics GmbH, Mannheim, Germany). Thyroid hormone levels including TSH, fT3, and fT4 were measured by electrochemiluminescence immunoassay method (Roche Diagnostics GmbH, Mannheim, Germany). Fasting plasma glucose (FPG) was measured by the glucose oxidase method (Olympus AU 2700, Beckman Coulter Inc., Brea, CA, USA). Insulin levels were measured with an immunoradiometric assay (IRMA) (Immunotech, Prague, Czech Republic). Serum VEGF levels were determined by the enzyme-linked immunosorbent assay method using a commercially available kit (R&D Systems, Minneapolis, MN, USA). The average intra- and interassay CVs for VEGF were ≤6.7% and ≤8.8%, respectively. Homeostatic model assessment for insulin resistance (HOMA-IR) was calculated according to the following formula: glucose (mg/dL) × insulin ([µIU/mL])/405. 

### 2.3. Statistical analysis

Statistical analyses were carried out using SPSS 17.0 (SPSS Inc., Chicago, IL, USA). Differences between means were analyzed by Wilcoxon rank test and paired sample t-test according to the normality of distribution determined by Kolmogorov–Smirnov test. Correlation analysis was performed with Spearman correlation rank test. The results are given as mean ± standard deviation or median (range) when appropriate, and P < 0.05 was accepted as statistically significant. Changes in the thyroid volume, dominant thyroid nodule volume, and sum of all nodule volumes were correlated with changes in BMI, VEGF, TC, LDL-C, and HOMA-IR by generalized estimated equation (GEE) analysis. 

## 3. Results

A total of 69 patients (21 males, 48 females) with dyslipidemia (mean age: 55.2 ± 8.6 years) were included in the study; 43 patients were treated with atorvastatin and the remaining 26 patients were treated with rosuvastatin. In patients with thyroid nodules, 26 patients were on atorvastatin treatment and 11 patients were on rosuvastatin treatment. The average dosages for atorvastatin and rosuvastatin were 13.5 ± 4.8 mg and 10.9 ± 3.0 mg, respectively. In patients without thyroid nodules, 17 patients were on atorvastatin therapy and 15 patients were on rosuvastatin treatment; the average dosages were 12.4 ± 4.4 mg and 11.3 ± 3.5 mg, respectively. Atorvastatin and rosuvastatin dosages were similar in patients with and without thyroid nodules (13.5 ± 4.8 vs. 12.4 ± 4.4 mg, P > 0.05 for atorvastatin; 10.9 ± 3 vs. 11.3 ± 3.5 mg, P > 0.05 for rosuvastatin). Patients with and without thyroid nodules had similar metabolic parameters and serum VEGF levels (Table 1). Additionally, incidence of type 2 diabetes and hypertension was similar among the groups along with medications taken, including antihypertensive and antidiabetic drugs (Table 2). Thyroid volume and dominant thyroid nodule volume were similar in patients with and without diabetes (13.2 ± 6.2 vs. 11.4 ± 8.2 mL, P = 0.329 and 0.68 ± 1.2 vs. 0.58 ± 1.1 mL, P = 0.821, respectively) and in patients with and without hypertension (14.3 ± 7.9 vs. 11.2 ± 6.1 mL, P = 0.09 and 0.63 ± 1.1 vs. 0.63 ± 1.4 mL, P = 0.998, respectively) . 

**Table 1:** Metabolic parameters of patients with and without thyroid nodules.

	Nodule (–)n = 32	Nodule (+)n = 37	P
Age (years)	53.2 ± 9.6	56.8 ± 7.3	0.736
Sex (male/female)	13/19	9/28	0.139
BMI (kg/m2)	30.2 ± 4.5	30.9 ± 4.5	0.489
WHR	0.90 ± 0.07	0.92 ± 0.08	0.219
SBP (mmHg)	132.4 ± 18.9	132.0 ± 23.7	0.932
DBP (mmHg)	82.6 ± 12.3	84.1 ± 18.9	0.712
TSH (µIU/mL)	3.01 ± 2.61	2.35 ± 1.15	0.090
Total cholesterol (mg/dL)	240.9 ± 40.5	236.4 ± 33.2	0.612
Triglyceride (mg/dL)	145.6 ± 67.5	153.6 ± 53.9	0.586
LDL-C (mg/dL)	159.1 ± 38.9	155.8 ± 22.4	0.655
HDL-C (mg/dL)	53.2 ± 14.2	53.0 ± 16.0	0.958
FPG (mg/dL)	114.7 ± 30.1	111.6 ± 34.7	0.698
Insulin (µIU/mL)	13.3 ± 6.7	11.2 ± 5.5	0.146
HOMA-IR	3.8 ± 2.4	3.1 ± 1.7	0.148
VEGF (pg/mL)	562.9 ± 291.0	513.9 ± 229.1	0.436

Data are expressed as mean ± SD. BMI: Body mass index, WHR: waist to hip ratio, SBP: systolic blood pressure, DBP: diastolic blood pressure, LDL-C: low-density lipoprotein cholesterol, HDL-C: high-density lipoprotein cholesterol, FPG: fasting plasma glucose, HOMA-IR: homeostatic model assessment for insulin resistance, VEGF: vascular endothelial growth factor.

**Table 2:** Characteristics of patients with and without thyroid nodules.

	Nodule (–)n = 32	Nodule (+)n = 37	P
Diabetes, % (+ / –)	71.1 (23 / 9)	52.3 (19 / 18)	0.091
Hypertension, % (+ / –)	31.4 (10 / 22)	48.1 (18 / 19)	0.219
Smoking, % (+ / –)	25.1 (8 / 24)	16.2 (6 / 31)	0.601
ARB, % (+ / –)	18.8 (6 / 26)	18.9 (7 / 30)	0.986
ACE inh, % (+ / –)	12.5 (4 / 28)	21.7 (8 / 29)	0.319
Beta blocker, % (+ / –)	6.2 (2 / 30)	13.5 (5 / 32)	0.310
OAD, % (within diabetics)	60.9 (14 / 23)	6.2 (12 / 19)	0.612
Insulin, % (within diabetics)	30.4 (7 / 23)	36.8 (7 / 19)	0.586

ARB: Angiotensin receptor blocker, ACE inh: angiotensin-converting enzyme inhibitor, OAD: oral antidiabetic drugs.

 Clinical and laboratory characteristics of the study patients without and with thyroid nodules at baseline and at the end of 6 months of therapy are shown in Tables 3 and 4, respectively. Anthropometric measurements, systolic and diastolic blood pressures, thyroid function tests, thyroid autoantibody levels, and glucose metabolism parameters remained unchanged after therapy. Median thyroglobulin level in all patients was 8.11 (0–186) ng/mL and its level was unchanged during the study period (8.33 [0–114] ng/mL) (P = 0.626). As expected, TC, LDL-C, and triglyceride levels decreased significantly after statin therapy. Serum VEGF levels did not change after statin treatment in patients with thyroid nodules (517.6 ± 231.1 vs. 521.0 ± 253.6 pg/mL, P = 0.931). Statin treatment resulted in a decrease in serum VEGF levels in patients without nodules (556.9 ± 288.2 vs. 508.7 ± 256.9 pg/mL, P = 0.030) (Table 3). 

**Table 3:** Clinical and biochemical features of patients without thyroid nodules at baseline and at 6th month of therapy.

	Baselinen = 32	6th monthn = 32	P
Age (years)	55.2 ± 8.6		
Sex (male/female)	12 / 20		
BMI (kg/m2)	30.5 ± 4.5	30.3 ± 4.6	0.896
WHR	0.91 ± 0.08	0.90 ± 0.09	0.189
SBP (mmHg)	131.7 ± 19.7	131.5 ± 24.8	0.850
DBP (mmHg)	82.2 ± 12.2	79.9 ± 10.1	0.287
TSH (µIU/mL)	3.03 ± 2.57	2.52 ± 1.43	0.269
fT3 (pmol/L)	4.81 ± 0.79	4.79 ± 0.85	0.829
fT4 (pmol/L)	15.68 ± 1.89	16.46 ± 2.92	0.181
Anti-TPO (IU/mL)	25 (5–1000)	27 (5–1000)	0.750
Anti-TG (IU/mL)	20 (20–19,557)	20 (20–27,408)	0.187
Total cholesterol (mg/dL)	240.7 ± 40.2	163.1 ± 29.6	<0.001
Triglyceride (mg/dL)	145.7 ± 66.5	126.8 ± 48.0	<0.001
LDL-C (mg/dL)	158.5 ± 38.4	91.7 ± 23.5	<0.001
HDL-C (mg/dL)	53.3 ± 14.0	50.5 ± 15.9	0.040
FPG (mg/dL)	113.9 ± 29.5	116.5 ± 34.5	0.552
Insulin (µIU/mL)	13.2 ± 6.7	13.5 ± 8.5	0.799
HOMA-IR	3.4 ± 2.1	3.7 ± 2.5	0.113
VEGF (pg/mL)	556.9 ± 288.2	508.7 ± 256.9	0.030
Thyroid volume (mL)	12.40 ± 7.00	12.43 ± 7.33	0.854

Data are expressed as mean ± SD or median (range) as appropriate. BMI: Body mass index, WHR: waist to hip ratio, SBP: systolic blood pressure, DBP: diastolic blood pressure, LDL-C: low-density lipoprotein cholesterol, HDL-C: high-density lipoprotein cholesterol, FPG: fasting plasma glucose, HOMA-IR: homeostasis model assessment for insulin resistance, VEGF: vascular endothelial growth factor.

Treatment with statin for 6 months caused a significant decrease in the mean dominant thyroid nodule volume (P = 0.013) (Table 4). No difference was observed in thyroid volume before and after statin treatment. Sonographic characteristics of the evaluated nodules including structure, echogenicity, vascularity, border shape, and halo presence did not differ before and after therapy. Doppler ultrasonographic evaluation revealed no change in the vascularity of thyroid nodules.

**Table 4:** Clinical and biochemical features of patients with thyroid nodules at baseline and at 6th month of therapy.

	Baselinen = 37	6th monthn = 37	P
Age (years)	56.6 ± 7.4		
Sex (male/female)	9 / 28		
BMI (kg/m2)	30.7 ± 4.5	30.5 ± 3.9	0.395
WHR	0.91 ± 0.08	0.91 ± 0.07	0.821
SBP (mmHg)	131.7 ± 24.0	131.8 ± 29.6	0.585
DBP (mmHg)	84.1 ± 18.9	82.6 ± 13.7	0.993
TSH (µIU/mL)	1.96 ± 1.17	2.03 ± 1.06	0.733
fT3 (pmol/L)	4.65 ± 0.68	4.81 ± 0.68	0.353
fT4 (pmol/L)	15.70 ± 1.93	15.72 ± 2.20	0.896
Anti-TPO (IU/mL)	21 (7–1000)	25.5 (7–1000)	0.935
Anti-TG (IU/mL)	20 (20–143)	20 (20–189)	0.310
Total cholesterol (mg/dL)	237.1 ± 33.5	176.2 ± 36.0	<0.001
Triglyceride (mg/dL)	151.3 ± 56.0	119.5 ± 43.8	<0.001
LDL-C (mg/dL)	156.6 ± 22.0	100.8 ± 28.8	<0.001
HDL-C (mg/dL)	53.4 ± 16.7	53.9 ± 15.8	0.555
FPG (mg/dL)	111.3 ± 35.0	110.9 ± 26.7	0.735
Insulin (µIU/mL)	11.0 ± 5.6	13.2 ± 9.2	0.140
HOMA-IR	3.0 ± 1.7	3.6 ± 2.2	0.086
VEGF (pg/mL)	517.6 ± 231.1	521.0 ± 253.6	0.931
Thyroid volume (mL)	14.12 ± 7.21	14.3 ± 7.52	0.884
Dominant nodule volume (mL)	0.16 (0.01–6.09)	0.14 (0.01–6.44)	0.013
Total nodule volume (mL)	0.21 (0.03–6.09)	0.20 (0.01–7.96)	0.039

Data are expressed as mean ± SD or median (range) as appropriate. BMI: Body mass index, WHR: waist to hip ratio, SBP: systolic blood pressure, DBP: diastolic blood pressure, LDL-C: low-density lipoprotein cholesterol, HDL-C: high-density lipoprotein cholesterol, FPG: fasting plasma glucose, HOMA-IR: homeostasis model assessment for insulin resistance, VEGF: vascular endothelial growth factor.

Correlation of changes in the thyroid volume, dominant thyroid nodule volume, and sum of thyroid nodule volumes with changes in BMI, VEGF, TC, LDL-C, and HOMA-IR did not show any associations. Comparison of the changes in the studied parameters was similar between diabetic and nondiabetic patients as well as in hypertensive and normotensive patients. 

## 4. Discussion

The association of metabolic syndrome components and thyroid disorders is of clinical interest. It has been shown that serum levels of TSH are higher in obese patients than in healthy controls, supporting the hypothesis that the axis involving the hypothalamus, the pituitary, the thyroid, and the adipose tissue is somehow disrupted (24–27). Insulin is a thyroid growth factor that stimulates proliferation of thyroid cells in culture, and it has been shown that insulin resistance yields increased thyroid volume and nodularity in euthyroid women (1). Moreover, it has also been demonstrated that thyroid nodularity and thyroid volume were increased in metabolic syndrome where all the components including dyslipidemia were significantly associated with increased thyroid volume and thyroid nodularity (2,3). In our cohort of dyslipidemic patients, we aimed to evaluate the effect of statin treatment and correction of dyslipidemia on thyroid nodules, thyroid volume, and serum VEGF levels. We found that serum VEGF levels were similar in dyslipidemic patients with and without thyroid nodules. Although statin treatment for 6 months resulted in a decrease in serum VEGF levels in patients without thyroid nodules, no difference in serum VEGF levels was observed in patients with thyroid nodules. The dominant nodule volume decreased after statin therapy.

Studies have been done evaluating the effect of statins on the thyroid. In vitro studies have confirmed the apoptotic effects of statins on proliferating thyroid cells, as well as differentiated and anaplastic thyroid cancer cells (21,22,28). In addition, it has also been demonstrated that administration of lovastatin to rats inhibited thyroid hypertrophy and hyperplasia induced by goitrogenic agents (22). Although positive effects of statins on thyroid tissue have been demonstrated by a number of authors, including us, it must also be proven in the clinical arena. Capelli et al. carried out a study where they demonstrated that therapy with statins for at least 5 years was associated with significantly lower prevalence, number, and volume of thyroid nodules and smaller thyroid size (24). Capelli et al. found that although their patients were on statin therapy for 5 years, the duration of treatment was not related to statin’s effects on the thyroid gland, and thus they could not draw a conclusion on shorter periods of treatment. They evaluated the use of concomitant drugs that may potentially affect the thyroid gland, such as ACE inhibitors and beta blockers; however, there was no evaluation of insulin resistance, hyperglycemia, obesity, or hypertension. Chon et al. also conducted a similar study where they evaluated statin’s effect on thyroid nodules in patients with type 2 diabetes and demonstrated a decrease in nodule prevalence with statin treatment for at least 5 years, but their findings were less pronounced than the findings of Capelli et al. They suggested that this discrepancy could be explained by higher insulin resistance in their patients (28). Both studies evaluated the incidence of nodules under statin treatment. In our study, we evaluated the possible effect of statin treatment on existing nodules and found a decrease in dominant thyroid nodule volume even after 6 months of statin treatment; however, there was no change in thyroid volume. A number of confounding factors may influence this finding, such as the relative iodine deficiency in our study population, as Chon et al. explained. However, the most reasonable explanation for this finding is the metabolic disease background of the patients. In our dyslipidemic patient population including diabetic and hypertensive patients as well as nondiabetic and normotensive patients, statin treatment ameliorating one of the metabolic parameters may be effective in decreasing thyroid nodule volume. However, GEE analysis failed to show an association between change in lipid levels and thyroid nodule volume, suggesting a complicated cascade other than correction of dyslipidemia. 

Capelli et al. and Chon et al. retrospectively evaluated the prevalence of thyroid nodules in statin-treated patients and compared the results with those of statin-naïve controls. Due to the nature of their study designs, there is no potential explanation for how statin resulted in a decrease in thyroid nodularity. Vascularization is an important feature of tumor growth that might contribute to the growth and progression of thyroid nodules and VEGF is one of the most powerful angiogenesis stimulators (4,29). High expression of VEGF in thyroid cancer was found to be correlated with advanced tumor stage, lymph node metastasis, and increased risk of recurrence (5–7). Therefore, VEGF inhibitors are approved for the treatment of advanced thyroid cancers (30). VEGF has also been found to be upregulated in the pathogenesis of benign thyroid disorders (9). Increased expression of VEGF and its receptors in nodular tissue of uninodular and recurrent goiter as well as in the entire tissue of multinodular goiter has been demonstrated (29). Furthermore, tyrosine kinase inhibitors may result in hypothyroidism, indicating a potential role for VEGF in signaling even in normal thyroid tissue (30). However, controversial results have been found related to serum VEGF levels and thyroid nodule formation (11–14). These discrepancies may be explained by the evaluation of a group of patients of mixed metabolic disease statuses in these studies, complicating the evaluation of serum VEGF levels, as serum VEGF levels are induced by insulin and hyperglycemia (4,17). In our study, patients with and without thyroid nodules had similar incidences of diabetes, hypertension, angiotensin-converting enzyme inhibitors, angiotensin receptor blockers, beta blockers, oral antidiabetic drugs, and insulin use, as well as smoking history. In dyslipidemic patients with an unfavorable metabolic profile, patients with and without thyroid nodules had similar VEGF levels. Hence, after excluding the bias of metabolic profile, serum VEGF levels did not demonstrate an association with nodule formation.

The results of several in vivo studies are consistent with the idea that statins exert antiangiogenic activities, especially via VEGF-dependent pathways (31,32). A number of studies demonstrated a reduction in VEGF levels in patients with dyslipidemia treated with statins (17,33,34). The dose-dependent effect of statins on angiogenesis has not been elucidated. Based on experimental studies, some authors suggested that low doses of statin may have proangiogenic effects (35). Statins can promote angiogenesis in ischemic tissue via the increase of endothelial progenitor cells. However, studies in diabetic and hypercholesterolemic patients have demonstrated that low doses also reduce serum VEGF levels (17,33). VEGF plays a crucial role among proangiogenic factors. In our study, low-dose statin resulted in a decrease in VEGF levels in patients without thyroid nodules, demonstrating the antiangiogenic effect of the statins. However, in patients with thyroid nodules, statin treatment did not alter serum VEGF levels, supporting neither proangiogenic nor antiangiogenic effects of statins on these patients. The lowering effect of statins on serum VEGF levels was blunted in patients with thyroid nodules. Metabolic conditions as well as thyroid nodule presence, which may also be related to metabolic conditions, seem to have an influence on serum VEGF levels. The presence of thyroid nodules may possibly have a subtle influence on serum VEGF levels where serum VEGF levels are not altered but fail to respond to exogenous stimuli such as statin treatment. 

In conclusion, 6-month statin treatment decreases dominant thyroid nodule volume and total nodule volumes. Although statin treatment decreases serum VEGF levels in dyslipidemic patients without thyroid nodules, it has no lowering effect on serum VEGF levels in patients with thyroid nodules. The decrease in thyroid nodule volume by statin treatment was associated with neither metabolic parameters nor serum VEGF levels. Our results confirm the complex interaction between thyroid nodule formation and metabolic parameters, as all of our patients had dyslipidemia, but the ones with nodules failed to respond to statin treatment with decreasing VEGF levels. Local expression of VEGF within thyroid tissues and nodules may be of potential interest in further studies to elucidate whether VEGF plays a significant role in statin-associated changes in thyroid/nodule volume.

## References

[ref0] (2008). the thyroid gland as another victim of the insulin resistance syndrome. Thyroid.

[ref1] (2007). Thyroid function is associated with components of the metabolic syndrome in euthyroid subjects. J Clin Endocrinol Metab.

[ref2] (2009). Metabolic syndrome and its components are associated with increased thyroid volume and nodule prevalence in a mild-to-moderate iodine-deficient area. Eur J Endocrinol.

[ref3] (2015). The association between insulin resistance and vascularization of thyroid nodules. J Clin Endocrinol Metab.

[ref4] (2011). Sipal S. Correlation of E-cadherin, VEGF, COX-2 expression to prognostic parameters in papillary thyroid carcinoma. Exp Mol Pathol.

[ref5] (2011). VEGFR1 expression is related to lymph node metastasis and serum VEGF may be a marker of progression in the follow-up of patients with differentiated thyroid carcinoma. Eur J Endocrinol.

[ref6] (2001). Intensity of vascular endothelial growth factor expression is associated with increased risk of recurrence and decreased disease-free survival in papillary thyroid cancer. Surgery.

[ref7] (1995). Stimulation by thyroid-stimulating hormone and Grave’s immunoglobulin G of vascular endothelial growth factor mRNA expression in human thyroid follicles in vitro and flt mRNA expression in the rat thyroid in vivo. J Clin Invest.

[ref8] (2005). Angiogenesis in benign and malignant thyroid disease. Thyroid.

[ref9] (1999). Expression of angiogenesis stimulators and inhibitors in human thyroid tumors and correlation with clinical pathological features. Am J Pathol.

[ref10] (2002). Expression of vascular endothelial growth factor family messenger RNA in diseased thyroid tissues. Surg Today.

[ref11] (2013). Vascular endothelial growth factor marker for proliferation in thyroid diseases?. Exp Clin Endocrinol Diabetes.

[ref12] (2010). Expression of vascular endothelial growth factor and presence of angiovascular cells in tissues from different thyroid disorders. World J Sur.

[ref13] (2008). Therapeutic potential of statins in thyroid proliferative disease. Nat Clin Pract Endocrinol Metab.

[ref14] (2007). The inflammation hypothesis and its potential relevance to statin therapy. Am J Cardiol.

[ref15] (2007). Statins and the vascular endothelial inflammatory response. Trends Immunol.

[ref16] (2014). Statins in low doses reduce VEGF and bFGF serum levels in patients with type 2 diabetes mellitus. Pharmacology.

[ref17] (2012). Prospective, randomized, single-blind comparison of effects of 6 months’ treatment with atorvastatin versus pravastatin on leptin and angiogenic factors in patients with coronary artery disease. Heart Vessels.

[ref18] (2012). Atorvastatin reduces vascular endothelial growth factor (VEGF) expression in human non-small cell lung carcinomas (NSCLCs) via inhibition of reactive oxygen species (ROS) production. Mol Oncol.

[ref19] (1999). Inhibition of farnesylation blocks growth but not differentiation in FRTL-5 thyroid cells. Biochimie.

[ref20] (1999). Prenyltransferase inhibitors induce apoptosis in proliferating thyroid cells through a p53-independent CrmA-sensitive, and caspase-3-like protease-dependent mechanism. Endocrinology.

[ref21] (2006). HMG-CoA reductase inhibitors inhibit rat propylthiouracil-induced goiter by modulating the ras-MAPK pathway. J Mol Med.

[ref22] (2011). Rosuvastatin induces apoptosis in cultured human papillary thyroid cancer cells. J Endocrinol.

[ref23] (2008). Reduced thyroid volume and nodularity in dyslipidaemic patients on statin treatment. Clin Endocrinol (Oxf).

[ref24] (2003). The effect of body weight and weight loss on thyroid volume and function in obese women. Clin Endocrinol (Oxf).

[ref25] (2006). Thyroid function in humans with morbid obesity. Thyroid.

[ref26] (2007). Free triiodothyronine and thyroid stimulating hormone are directly associated with waist circumference, independently of insulin resistance, metabolic parameters and blood pressure in overweight and obese women. Clin Endocrinol (Oxf).

[ref27] (2011). Influence of long-term statin use in type 2 diabetic patients on thyroid nodularity in iodine-sufficient area. Exp Clin Endocrinol Diabetes.

[ref28] (2007). Vascular endothelial growth factor (VEGF), VEGF receptors expression and microvascular density in benign and malignant thyroid diseases. Int J Exp Pathol.

[ref29] (2011). Vandetanib (ZD6474) in the treatment of medullary thyroid cancer. Clin Med Insights Oncol.

[ref30] (2007). A novel tyrosine-kinase selective inhibitor, sunitinib, induces transient hypothyroidism by blocking iodine uptake. J Clin Endocrinol Metab.

[ref31] (2010). Atorvastatin inhibits inflammatory angiogenesis in mice through down regulation of VEGF, TNF-α and TGF-β1. Biomed Pharmacother.

[ref32] (2006). Simvastatin reduces serum level of vascular endothelial growth factor in hypercholesterolemic patients. J Cardiovasc Pharmacol.

[ref33] (2006). Vascular endothelial growth factor serum concentrations in hypercholesterolemic patients. Scand J Clin Lab Invest.

